# Identification of nutritionally modifiable hormonal and epigenetic drivers of positive and negative growth deviance in rural African fetuses and infants: Project protocol and cohort description

**DOI:** 10.12688/gatesopenres.13101.1

**Published:** 2020-02-24

**Authors:** Sophie E. Moore, Andrew M. Doel, Ken K. Ong, David B. Dunger, Nabeel A. Affara, Andrew M. Prentice, Robin M. Bernstein, HERO-G Working Group

**Affiliations:** 1 Department of Women and Children's Health, King's College London SE1 1UL, London, UK; 2 MRC Unit The Gambia, London School of Hygiene & Tropical Medicine, Fajara, The Gambia; 3 Department of Paediatrics, University of Cambridge Addenbrooke's Hospital Cambridge, Cambridge, UK; 4 Department of Pathology, University of Cambridge, Cambridge, UK; 5 Department of Anthropology, University of Colorado, Boulder, CO, 80309, USA; 6 Health and Society Program, Institute of Behavioral Science, University of Colorado, Boulder, CO, USA

**Keywords:** Growth, epigenetics, hormones, infants, The Gambia, stunting, wasting

## Abstract

Growth retardation (stunting, wasting and poor organ development) among children in low-income countries has major short and long-term health consequences yet very little is known about the nutritional and environmental influences on the key hormonal axes regulating child growth in these settings, nor the tempo and timing of faltering episodes. Here we describe the study protocol and provide a cohort description of the Hormonal and Epigenetic Regulators of Growth (HERO-G) study. This prospective cohort study from rural Gambia, West Africa, followed mothers and children longitudinally from pre-conception, through pregnancy, delivery, and to two years of child age

A total of 251 eligible mother-infant pairs were recruited into the HERO-G study, with 206 (82%) followed up until two years of age. Women were seen at scheduled antenatal appointments at 20, 28 and 36 weeks of gestation, and at delivery, where possible. Between one week and 12 months of age, infants were visited every second day for collection of detailed anthropometry and morbidity data. Infants identified as about to enter a growth faltering episode at these visits entered a more detailed 20-day protocol, with the collection of dried blood spots, anthropometry and body composition. All infants were seen for scheduled clinic visits at 3, 6, 9, 12, 18 and 24 months of age for clinical examination and venous blood draw.

Data from the HERO-G study is being used to explore three major mechanistic pathways influencing growth: 1) genome-wide investigations for signatures of epigenetic effects on any loci that might affect growth; 2) frequent anthropometric measurement coupled with non-invasive monitoring for rapid identification and interrogation of real-time faltering patterns and aetiology; 3) focused measurement of hormones and cytokines that act together in an integrated manner, both *in utero* and after birth, to coordinate patterns of growth with immune activation, inflammation, and nutritional status.

## Introduction

Growth retardation (stunting, wasting and poor organ development) among children in low-income countries is a major contributor to morbidity and mortality, has intergenerational sequelae and limits the growth of human capital ^1^. Yet, remarkably, almost nothing is known about the nutritional and environmental influences on the key hormonal axes regulating child growth in these settings. Growth and development in the first 1000 days from conception requires a complex orchestration that represents an interplay between an individual’s genetically and epigenetically determined growth trajectories and the environment. Achievement of different ‘size milestones’ (e.g., doubling of birthweight) can vary markedly within and across populations based on individual navigation of optimal outcomes in response to differing nutritional environments and disease ecologies. Similarly, there can be wide variations in the composition of tissue gained. Hormones, as key mediators of growth, are responsive to nutrition and infection, and their expression has been demonstrated to be epigenetically regulated both pre- and postnatally, in many cases in response to maternal health and nutrition before conception and during gestation ^2^. Several key hormone systems exert multiple effects on appetite, growth, metabolism and immune function and respond to stimuli in early life to determine ‘set points’ that in turn function as pacesetters during early growth. Current knowledge of how these systems develop and how they affect growth-related physiology in response to pre- and post-natal experience is largely derived from work with model animals or from clinical studies of disease syndromes; virtually nothing is known from populations in resource poor settings.

Rural Gambian infants are small at birth relative to international standards, show positive growth during the first few months of life, and then enter a period of reduced growth marked by profound faltering up until 24 months of postnatal life ^3, 4^. Earlier attempts to explain these patterns, which are shared by many populations across the developing world to varying degrees, centred solely on malnutrition. Subsequent studies focused on disease and infection as primary causes of growth retardation, via chronic stimulation of immune and inflammatory systems in infants who encounter challenges to recovery in chronic and recurrent episodes. Intestinal damage, in particular, has been implicated as largely responsible for growth faltering in these populations; however, establishing clear cause-and-effect relationships remains elusive. Previous studies, which measured infant growth, measures of intestinal permeability, and circulating markers of inflammation were able to suggest a strong negative effect of intestinal enteropathy on growth ^5, 6^ – but the frequency of sampling and anthropometric measurements did not capture the precise sequence of events during episodes of faltering and recovery, critical for establishing a chain of causality.

Here we present the study protocol and cohort description for the Hormonal and Epigenetic Regulators of Growth (HERO-G) study. This prospective cohort followed mothers and children in rural Gambia longitudinally from pre-conception, through pregnancy, delivery and to two years of child age with the primary focus of exploring three main mechanistic pathways influencing growth: 1) genome-wide investigations for signatures of epigenetic effects (methylation) on any loci that might affect growth or are associated with disease processes; 2) frequent anthropometric measurement coupled with non-invasive monitoring for rapid identification and interrogation of real-time faltering patterns and aetiology; 3) focused measurement of hormones and cytokines that act together in an integrated manner, both *in utero* and after birth, to coordinate patterns of growth with immune activation, inflammation, and nutritional status.

## Protocol

### Population and setting

The study was conducted in the West Kiang region of The Gambia, a rural subsistence farming community of savanna and farmland, within a roughly 750km ^2^ rectangular tract of farmland bounded on 3 sides by the River Gambia and its tributaries. Owing to a pronounced seasonality, food availability fluctuates widely across the year and the wet season, lasting from July to October, is a ‘lean’ period because stored staple foods from the previous year’s harvest are nearly depleted. At the same time, adults have an increased workload to prepare for the current year’s harvest. Conversely, the dry/harvest season (November to June) is a time of relative plenty with less agricultural activity, although many women tend to small village gardens during this time. More broadly, seasonal factors in this environment contribute to many aspects of variability in nutrition, health and behavior in both adults and children ^7, 8^.

### Sample selection and participant numbers

We aimed to recruit a cohort of 200 newborns, to follow longitudinally from pregnancy through to two years of age. Based on the assumption that ~25% of the cohort would be stunted by two years of age (length-for-age z-score < 2SD below the WHO reference), and that children would show characteristic patterns of growth over the first two years of life, including upward and downward centile crossing ^4^, this number was selected to enable exploratory analyses of the micro-growth patterns leading to stunting and the associated hormonal variations in this context but was not based on any formal power calculation.

The HERO-G study recruited participants from 18 of the 36 villages currently registered within the West Kiang Demographic Surveillance System (DSS; total resident population approximately 15,000) ^9^. Villages were selected based on size (larger villages) and proximity to the Medical Research Council (MRC) Keneba field station (villages had to be < 1 hours’ drive from Keneba). Following approval from the local community, all women aged between 18 to 45 years and resident in the selected villages were invited to participate, and written informed consent obtained. Trained field assistants explained the full details of the study to potential participants, covering all aspects of the study as laid out in the Information Sheet. Illiterate women had the full Information Sheet read to them in their local language; literate women were allowed to read the Information Sheet in their own time. Women were given an opportunity to consider their involvement in the project or speak to family members. Interested women were then asked to sign (or thumb print) the consent form.

Once consented, participating women were visited monthly by a trained member of the study team with a short questionnaire on the date of their last menstrual period (LMP) (Extended data ^10^). When a menses was reported as missed, a urine sample was collected for pregnancy testing using hCG tests (QuickVue™ One-Step hCG Urine Test, Quidel Corporation, UK, Catalog Number 20109). Women with a positive test were then invited to the clinic at MRC Keneba for an ultrasound examination. Women confirmed as being <28 weeks pregnant by ultrasound (Siemens ACUSON Antares Ultrasound Imaging System (Siemens Medical Solutions USA, Inc., California, USA with a CH6-2 (5.71 MHz) transducer) then entered the full HERO-G protocol (details below). Women’s weight (details below) was also recorded at each monthly home visit and a fingerprick sample of blood was collected and used to prepare dried blood spots (DBS) (Whatman 903 protein saver card, Whatman, GE Healthcare, US, Catalog Number 10534320).

### Study measurements

Pregnancy: Depending on gestational stage at the time of pregnancy confirmation, women were seen on at least three occasions over the course of pregnancy. Women who were less than 20 weeks’ gestation were seen at ‘booking’ (first visit after pregnancy confirmation – variable gestational stage), and then at 20, 28 and 36 weeks’ gestation. If women were over 20 weeks pregnant, they were seen at booking, 28 and 36 weeks only. At each antenatal visit, a study midwife measured maternal anthropometry (weight, height, lower leg length, mid-upper arm circumference (MUAC) and triceps skinfold thickness). Maternal weight was measured to the nearest 100g using Seca 803 digital scales, with women in minimal clothing and barefoot. Height was measured to the nearest 0.1cm using a Leicester portable stadiometer (Chasmors Ltd, UK), with women in light clothing and with shoes and head dresses removed. Lower leg length was measured as the distance between the ankle and the top of the patella (kneecap) using a soft tape measure, with women seated on a chair and with the knee angle at 90 degrees, and to the nearest 0.1cm. Triceps skinfold thickness was measured to the nearest 0.1cm using a Holtain Tanner/Whitehouse skinfold calliper (Chasmors Ltd, London, UK). At these visits, blood pressure was also measured using an Omron 705IT digital Blood Pressure Monitor.

A 10mL sample of venous blood was collected, haemoglobin levels assessed (Medonic M-Series automated hematology analyzer (Boule Medical)) and the remaining blood processed and divided into plasma and serum aliquots and frozen at -70°C. Where possible, women were also asked to provide a stool sample for biobanking. Finally, and at the booking visit only, additional data were collected on obstetric history, and a number of sociodemographic factors including years of schooling and bednet usage.

At the week 28 visit, all women were additionally invited to undergo a 75g oral glucose tolerance test (OGTT) following an overnight fast of at least 12 hours duration. Measurements of blood glucose were recorded immediately prior to administration of the oral glucose load (75g *Dexola* or *Glucola* solution), and at one and two hours following glucose consumption. Capillary blood glucose levels were measured immediately after collection with an Accu-Chek® glucometer. Samples of venous blood were also collected, processed (centrifuged at 3000RPM for 10 minutes at 4°C) within an hour of collection, and aliquots of plasma frozen for subsequent analysis of plasma glucose using a Cobas® Analyzer (Roche). If OGTT results indicated a risk of glucose intolerance, women were referred for further consultation.

Women were also seen at home on a weekly basis for the recording of maternal morbidity, using a standardised form (Extended data ^10^). Women requiring additional medical care were referred to the clinic in Keneba for follow up.

Delivery and week 1 visits: In this community, the majority of women choose to deliver in their homes with the support of a Traditional Birth Attendant (TBA). To help ensure deliveries were attended by a member of the HERO-G field team, trained field assistants were posted to each of the study villages and women (and their families) were asked to notify the field assistant once labour commenced. Following delivery, the field assistant collected a sample of cord blood (up to 15mL) and the placenta was carefully packed on ice. These samples were then transported to the MRC Keneba laboratory for processing. Cord blood samples were immediately processed, and aliquots of plasma and serum frozen for subsequent analysis.

Once in the laboratory, placental samples were processed for subsequent analysis as follows: One villous sample (~ 800mg) was collected from each of 4 sampling points, each at least 2 cm from the outer edge of the placenta, washed in cold PBS and then each divided into a further 8 x 100mg samples. Three samples from each location were then stored (within 10 minutes of processing) into RNAlater (Qiagen, Catalog Number 76106) for subsequent analysis. The remaining 5 samples from each site were snap frozen in liquid nitrogen and stored at −80°C for later analysis. A further wedge-shaped section from the edge of the placenta (~ 2cm x 2cm) was sampled from two different areas of the placenta and immersed in 10% buffered formalin for 12–24 hours. These samples were then washed in PBS and transferred to 70% ethanol, for subsequent histopathology. The remaining placenta was then weighed and measured.

Within 72 hours of delivery, all women and their newborn infants were then visited at home by one of the study midwives for a health check and neonatal examination. Infant anthropometry was measured (weight, length, MUAC, head circumference; see infant anthropometry section, below, for details). Mothers or neonates assessed as unwell were referred to the MRC Keneba clinic for follow up assessment.

In this community, it is traditional that infants are named on Day 7, with a traditional naming ceremony (Kulio) when, amongst other ceremonies, the infant’s hair is shaved. We used this opportunity to collect a sample of hair, for subsequent analysis of hair cortisol levels. Immediately following removal, an aliquot of the shaved hair was collected and stored in a paper envelope and kept at ambient temperature until shipment to the University of Colorado for subsequent analysis. This aliquot represented a mixture of hair from across the scalp in seven day old infants, mostly grown *in utero*. Approximately 10g of hair was weighed and placed into 2mL polypropylene tubes. Hair was washed twice with isopropanol, dried under a stream of air in a fume hood for 48 hours, then ground with one stainless steel ball per tube for 10 min at 25mHz, using a Retsch 400MM ball mill (Verder Scientific, Newton, PA, USA). Ground samples were incubated with 1 mL of HPLC-grade methanol on a rotating platform overnight at 170 x g. The next day, samples were centrifuged for 12 min at 4200 x g, and 875 mL of supernatant was removed and transferred to a new 2mL tube. The supernatant was dried down under a constant stream of nitrogen gas for ~15 min using a Micro-Vap system (Organomation, Berlin, MA, USA). Samples were then reconstituted with 0.5 mL assay diluent (packaged with kit, below), and reconstituted samples were assayed using a commercially available salivary cortisol kit (Salimetrics, State College, PA, USA, Catalog Number 1-3002), previously validated for use with human hair samples ^11^.

Infancy and early childhood; alternate day measurements: Between Day 7 and one year of age, infants were visited at home every second day for the collection of infant anthropometry. At each of these visits, data on infant weight, length, head circumference, MUAC, lower leg length, and triceps skinfold thickness were recorded and entered directly into a tablet-based electronic data capture system, using a study specific database. Measurements were made in triplicate, but not successively; i.e. rather than weight, weight, weight, length, length, length etc. measurements were made in singleton for each anthropometric parameter (weight, length, head circumference etc.) and then the whole sequence repeated twice over. Between each series of measurements the data entry screen was refreshed, so previous measurements could not be viewed by the assessor. This method was selected to try and ensure accurate replicate measurements, with as little bias from the assessor as possible.

All anthropometric measurements were made using regularly calibrated equipment and following standard protocols. Infant recumbent length was measured using a Seca 417 length board, to a precision of 0.1cm. Infant weight was taken with the infant naked, where possible, or wearing minimal clothing and using a Seca 336 digital weighing scale. Weights were recorded to the nearest 10g. Knee-heel length was measured using a custom-made knee-heel rod (Chasmors Ltd, London, UK), with precision to the nearest 0.1mm. MUAC was measured using flexible measuring tape (Seca 212) to the nearest 0.1mm. Head circumference was measured using a Seca 201 circumference measuring tape, to the nearest mm. Triceps skinfold thickness was measured using a Holtain Tanner/Whitehouse skinfold caliper (Chasmors Ltd, London, UK), to the nearest 0.1cm.

To further minimise intra- and inter-observer variation with the anthropometric measurements, full, comprehensive training was given to each field assistant at the start of the study and regular standardisation exercises were performed. The results of the anthropometry standardisation are presented below in Table 1 and Table 2.

**Table 1.** Summary statistics for 2015 anthropometry standardization.

Measurement	% inter-individual measurements made with acceptable precision	Absolute Inter-evaluator TEM range	Average Relative TEM	Average Reliability Index
Weight	86%	0.07–0.35 kg	0.31%	0.65
Body length	60%	0.44–0.89 cm	0.85%	0.31
Triceps skinfold	31%	0.46–1.13 cm	8.67%	0.38
Knee-heel length	66%	0.17–0.62 cm	1.81%	0.37
MUAC	74%	0.19–0.79 cm	3.40%	0.28
Head circumference	86%	0.20–0.53 cm	0.62%	0.47

MUAC - mid-upper arm circumference

**Table 2.** Summary statistics for 2016 anthropometry standardization.

Measurement	% inter-individual measurements made with acceptable precision	Absolute Inter-evaluator TEM range	Average Relative TEM	Average Reliability Index
Weight	100%	0.001–0.02kg	0.61%	1.26
Body length	71%	0.68–4.90 cm	0.85%	0.96
Triceps skinfold	52%	0.18–3.83 mm	9.96%	0.43
Knee-heel length	75%	0.02–1.31 cm	2.13%	1.00
MUAC	77%	0.04–0.88 cm	2.94%	0.60
Head circumference	74%	0.04–1.86 cm	1.11%	0.29

MUAC - mid-upper arm circumference

**Figure 1.**
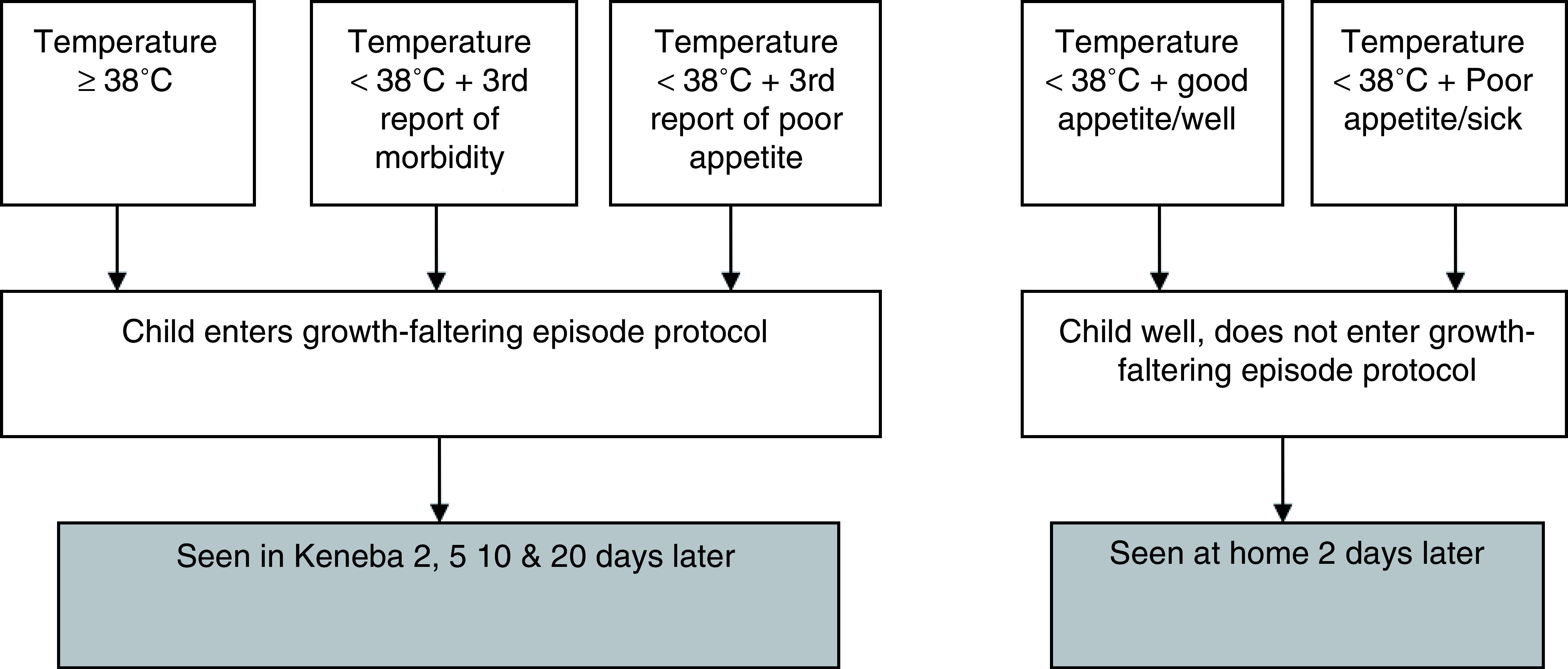
Decision tree for growth faltering episodes.

At the alternate day home visits, maternal reports on infant morbidity and appetite were collected, and the infant’s temperature was recorded. These data were entered into a “decision tree” ( Figure 1), as a means to capture an incipient growth faltering episode. Qualifying infants were flagged as high risk for faltering and were entered into an additional protocol (see 'Growth faltering protocol', below).

Infancy and early childhood clinic visits: When the infants were 3, 6, 9, 12, 18 and 24 months of age they were additionally seen at the MRC Keneba field station for a more detailed assessment. At these visits, infant anthropometry was measured as at the home visits, but with the addition of chest circumference, abdominal circumference, biceps, suprailiac and subscapular skinfolds. Infants who weighed <8kg at these visits had additional body composition measurements taken using the PEA POD Air Displacement Plethysmography system (COSMED srl, Italy). 

At each clinic visit, a venous blood sample was collected from each infant, Hb measured (as previous) and the remaining sample processed into plasma and serum aliquots for storage and subsequent analysis. Mothers were also asked to hand express a 10mL breast milk sample, which was subsequently split into smaller aliquots and frozen at -70°C. We collected all breastmilk samples at approximately the same time of day (13:00), and after the women had been provided with lunch. All samples were shipped frozen to University of Colorado where milk macronutrients were quantified using a LactoScope Fourier transform mid-infrared (FTIR) spectrophotometer (Delta Instruments B.V., Drachten, the Netherlands). Whole milk samples were thawed to room temperature, vortexed gently, and 2-mL aliquots of each sample were diluted 20× in 18 mL of doubly-distilled H _2_O. The diluted samples were warmed for 15 minutes to 38.5°C in a water bath prior to analysis. Concentrations of fat, total protein, true protein, lactose, and non-protein nitrogen concentrations in each milk sample were measured in triplicate.

In addition, prior to each clinic visit, mothers were provided with a stool collection kit and asked to collect a sample of infant stool, where possible. Stool samples were processed in the laboratory, and samples frozen at -70°C. At the visits at 3 and 12 months of infant age, additional saline wet preparations were prepared and also frozen for subsequent parasite analysis.

At the 3 and 12 month visits, an assessment of intestinal permeability was made using the shortened 2-hour dual sugar permeability test ^12^. Briefly, on arrival at the clinic, all infants were fitted with a urine collection bag and a pre-dosing sample of urine collected. Infants were then given a 2mL/kg dose of lactulose and mannitol solution (50mg/mL mannitol and 250mg/mL lactulose in the stock solution) and all further urine collected over the following 2 hours collected, and the total volume recorded. Aliquots of pre- and post-dose urine samples were then collected and frozen at -70°C until analysis.

At each clinic visit infants were also assessed by a study clinician, and health status recorded.

Growth faltering protocol: Infants who had either a single recording of a temperature of ≥38°C or a temperature of <38°C but either a third consecutive report of morbidity or low appetite entered a “growth faltering protocol”. The purpose of this protocol was to try to capture growth faltering episodes across a three-week window, so that body composition, hormonal and growth changes during this time could be studied in greater depth. Once identified, infants were scheduled to come to the MRC Keneba clinic on days 2, 5, 10, and 20 following identification of faltering.

At each visit, infants had anthropometric measurements as detailed previously, but with the addition of biceps, skinfold thickness, chest and waist circumferences. In addition, a fingerprick blood sample was collected and used to prepare DBS (as before). Additionally, for infants weighing <8kgs, body composition was measured by the PEA POD system, as described previously ^13, 14^. 

To reduce participant burden, each infant was seen for a faltering protocol a maximum of twice during their first year of life. Any infants <28 days of age were only seen for a health examination; no further “growth faltering” samples or measurements were taken. A summary of faltering events identified using this protocol is given in Table 3.

**Table 3.** Sex and season (of birth and of visit) of infants flagged for participation in the prospective faltering protocol.

Individuals with at least one faltering flag (N:148)	Individuals with two faltering flags (N: 51)
Male: 81 Female: 67	Male: 26 Female: 25
Dry season births: 99 Wet season births: 49	Dry season births: 35 Wet season births: 16
Falter in dry season: 54 Falter in wet season: 94	Falter in dry season: 23 Falter in wet season: 28

## Cohort description

**Figure 2.**
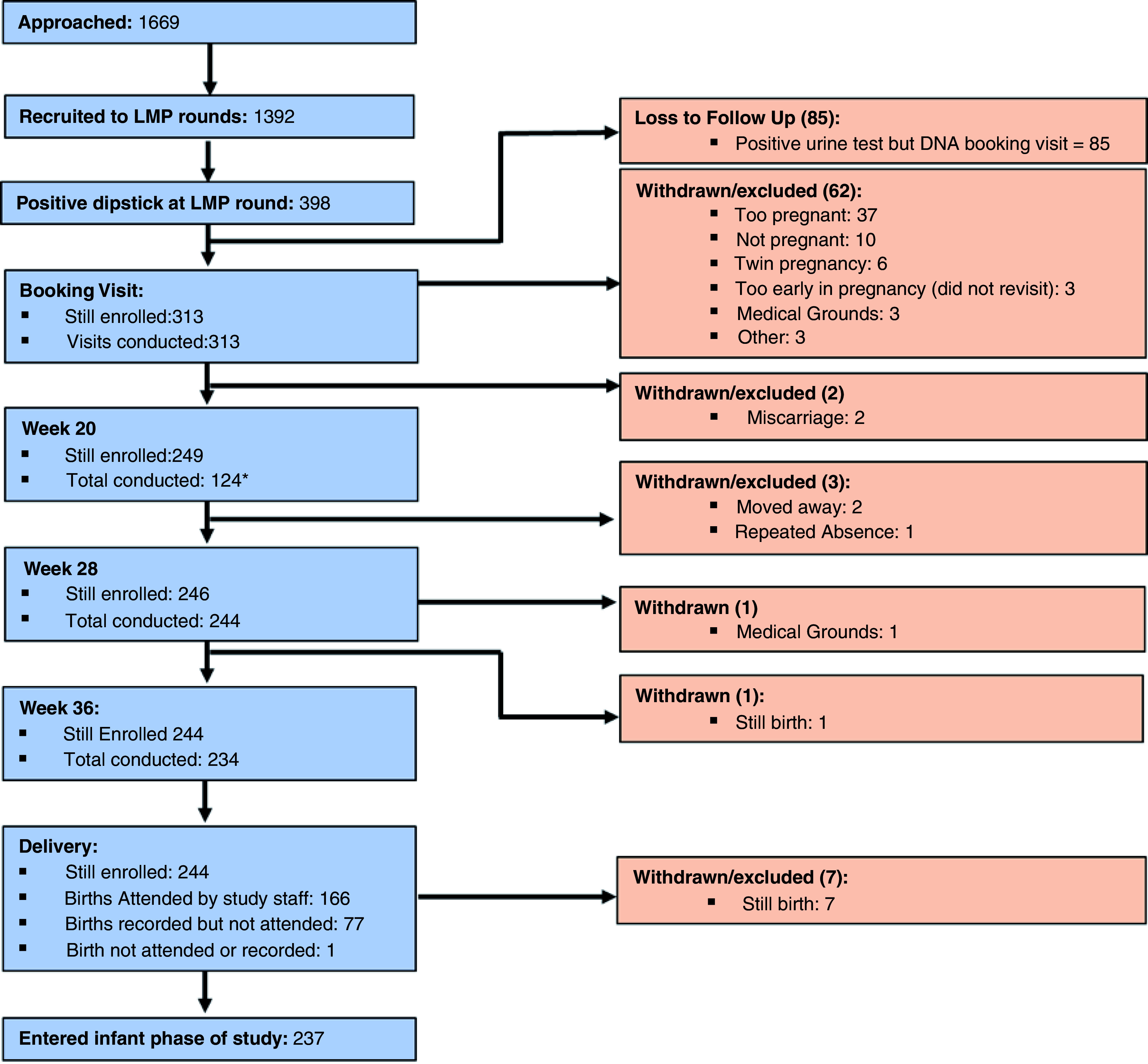
Participant flow chart; recruitment to delivery.

Figure 2 details the participant flow from recruitment to the delivery phase of HERO-G. Between March 2014 and March 2015, 1669 women were approached for inclusion in the study. 1392 women agreed to participate and were followed monthly for pregnancy surveillance. Over 15 months (July 2014 to October 2015) a total of 398 women produced a positive dipstick test. 313 of these women attended a booking visit at MRC Keneba and were confirmed as pregnant by ultrasound. At this visit, 62 women were found to be ineligible for further inclusion into the full HERO-G protocol; 37 women had a pregnancy beyond 28 weeks, 10 were not pregnant, 6 had a twin pregnancy, 3 were too early in pregnancy, 3 could not be included on medical grounds, 3 were excluded on undefined grounds, leaving 251 eligible women remaining for the remaining three antenatal visits (weeks 20, 28, and 36).

**Figure 3.**
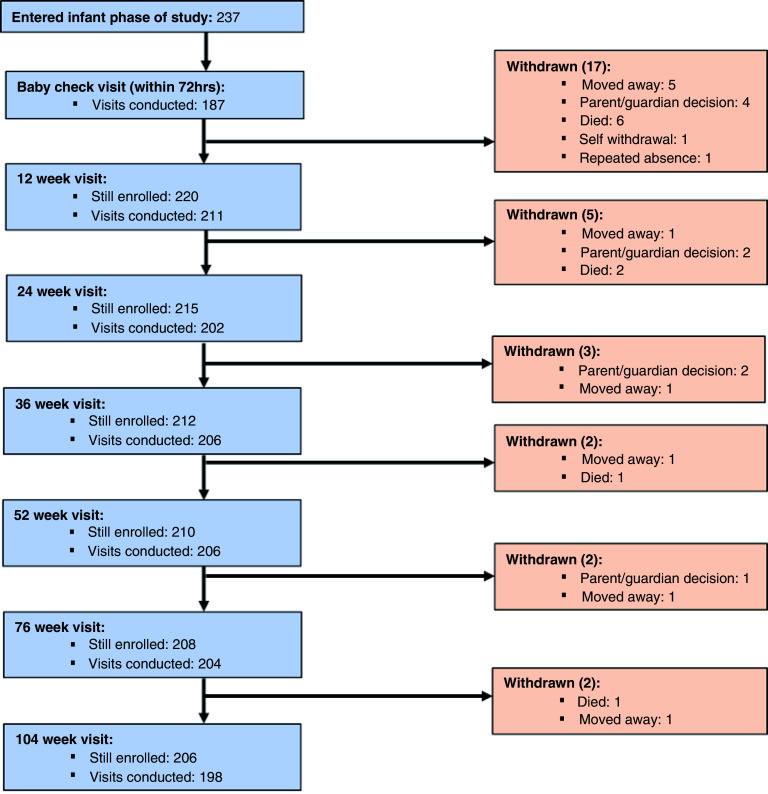
Participant flow chart; delivery to infant age 2 years.

Figure 3 details participant flow from delivery until final follow up when the infants reached two years of age. Of the 251 eligible pregnant women, 237 women delivered a live born infant and 166 of these births were attended by HERO-G study staff who collected a cord blood and placenta samples. Within 72 hours of delivery, a further ‘baby check’ was conducted on 187 infants. Between delivery and the end of the intense growth monitoring at one year of age, 27 infants were lost from the study. Reasons for loss to follow up included deaths ( *N*=9), families moved out of study area ( *N*=8), and 10 infants were withdrawn for other reasons. Between one and two years of age, a further 4 infants were withdrawn from the study. A total of 206 (82% of participants enrolled in the pregnancy phase; 87% of live born infants) remained in the study until two years of age, with data collected on 196 infants at the final point of follow up.

## Anthropometry standardisation results

Weight, body length, and head circumference were the most reliably measured anthropometric measurements, with average TEM between 0.31–1.11% across the 2015 and 2016 standardization exercises ( Table 2 and Table 3). Weight, body length, and head circumference were also the most precise measurements. In general, reliability, precision, and TEM improved between the 2015 and 2016 standardizations. This is likely due to the continued feedback to field workers from the supervisory team, as well as the increased rapport and familiarity between infants and field workers.

### Study ethics approvals

The HERO-G study was approved by the joint Gambia Government / MRC Unit The Gambia Ethics Committee (Project number SCC1313v3), and the University of Colorado Boulder Institutional Review Board (protocol number 13-0441).

### Data analysis and statistical plan

The high frequency anthropometric data collected in the HERO-G study allow for several analytical approaches to quantifying infant growth, including some replications and some novel methods. A brief overview of some examples of this planned work follows. Overall, HERO-G will provide detailed information concerning the patterns and processes that produce outcomes of interest (i.e., wasting, stunting). Rates of undernutrition within the cohort are detailed in Table 4.

**Table 4.** Rates of stunting, underweight and wasting in the HERO-G cohort.

Age	Stunting		Underweight		Wasting	
	Female	Male	Female	Male	Female	Male
3 months	8%	9%	6%	8%	2%	4%
6 months	10%	9%	4%	11%	3%	7%
9 months	9%	14%	9%	14%	4%	10%
12 months	10%	23%	12%	24%	11%	16.5%
18 months	18.5%	28%	19%	22%	10%	17%
24 months	15%	23%	23%	27%	13%	13%

Growth curve modeling. We modeled group-level (sex, season of birth, gestational age) HERO-G infant growth data using the Berkey-Reed model, but this resulted in over-smoothing of some interesting variation within these group-level trajectories. The SuperImposition by Translation And Rotation (SITAR) method for growth modeling ^15^ offers an advantage over traditional growth models since it does not assume any particular form of the curve ^16^. SITAR (available as the *sitar* (v. 1.1.1) package in R (R Core Team, 2019)) uses a spline to fit a mean curve then shifts each individual curve according to three coefficients (Size, Tempo, Velocity) to most closely match the mean function. We will use SITAR to model group-level HERO-G infant growth data.

Growth faltering. Growth faltering impacts populations in low- and middle-income countries across the world, particularly in contexts with a high burden of morbidity from infections. Although its negative impacts are widely recognized, detailed identification and quantification of faltering events has yet to be performed. This quantification could help to shape intervention strategies, particularly timing. We are developing a novel method for faltering event detection and quantification, which allows for the automatic detection of weight faltering in R (R Core Team, 2019), and the extraction of parameters regarding the faltering event such as the age of initiation and recovery, duration and depth of the faltering event.

Mini growth spurts. Previous approaches have modeled ‘mini growth spurts’ using the Gompertz function, and linked the number of these spurts to overall growth outcomes in linear dimensions ^17^. We will apply modifications of published Gompertz detection methods to quantify mini growth spurts in weight in the HERO-G infants, and assess the contribution of mini growth spurts to growth outcomes at one and two years of age.

Longer-term growth predictions from short-term data. We will replicate a method for determining the minimum number of total days of consecutive measurements at a given interval necessary to accurately predict longer-term patterns of growth rate ^18^. This method entails the calculation of half annual growth velocity, short-term growth velocity (growth in a dimension/every two days), and relative growth velocity as the short-term velocity divided by half annual growth velocity.

Infancy/childhood transition. A delay in the timing of the infancy-childhood transition has been proposed to contribute to sub-optimal growth outcomes ^19^. In well-nourished populations, the infancy-childhood transition (ICT) is estimated to occur between 9 and 12 months of age; in populations with marginal nutrition and high levels of morbidity, the ICT is estimated to take place as late as three years of age – or, a delayed ICT (DICT, ^20^). More recently, a method used to investigate the correlation structure of growth in infancy has offered a new way to identify the ICT ^21^, and this analysis suggests that this transition took place ~10–12 months of age in two cohorts of UK infants. We will apply this method to determine the age of ICT in HERO-G study infants.

Integration. There is very little comparative data on the nature of growth integration (i.e., the correlation across multiple dimensions over time) in infancy, especially from populations with high frequencies of morbidity and growth faltering. However, investigating growth integration in such populations offers a valuable window into the causes and consequences of tradeoffs during development, such as sparing the brain at the expense of the body. We will use velocity correlations and novel visualization methods to assess the degree of integration among body weight, body length, and head circumference growth velocities during the first year of life.

Synthesis. Outputs from laboratory and growth analyses will be modeled using a host of potential predictors (e.g., methylation patterns, metabolomic, hormonal, and inflammatory markers, intestinal permeability). In order to determine whether the hormones, methylation patterns, metabolomic profiles, or any of our other potential physiological predictors measured affect growth profiles, both overall and during episodes of faltering, the data need to be examined on multiple levels. First, in order to examine the overall relationship between hormone concentrations and category of growth deviance (positive or negative), while taking into account the multiple potentially important other factors (e.g., sex, maternal health and parity, breast milk nutritional components, season of birth), general linear mixed models will be constructed. Mixed models contain both fixed and random effects, and are particularly useful for repeated measures analysis because they have the capacity to estimate a number of different covariance structures. General linear mixed models (GLMM) are particularly appropriate since they adjust for the correlation between repeated measures of the same subject, a feature inherent to the longitudinal study design that has been proposed. GLMM that account for repeated anthropometric measures in infants will be used to examine the relationships between measures of growth and hormone concentrations. Similar models will be used to examine the relationships between episodes of faltering, specifically their degree and duration, and measures of body composition, hormone concentrations measured in samples taken during faltering episodes, metabolomic and lipidomic profiles, and results of lactulose: mannitol analyses. Separate analyses will be conducted if preliminary tests show that independent variables are correlated, and a Bonferroni correction will be used to account for the multiple comparisons. We will also employ functional principal components analysis (PCA) to identify patterns that characterize (e.g., 'growth archetypes'), and physiological correlates of, outcomes of interest ^22^.

### Dissemination of information

Local dissemination will occur via village meetings to feedback the overall study results to participants, village elders and other interested parties. The work will be presented in open seminars at MRC Unit The Gambia. The results will be shared with the Gambian National Nutrition Agency (NaNA) and the Gambian Government via the annual meeting. International dissemination will be through conference presentations and seminars at CU, Kings College and LSHTM. The primary avenue for dissemination will be through peer-reviewed publications in international journals. If any findings are deemed to be of broad public interest we will work with the press offices at CU, KCL, LSHTM and MRCG@LSHTM to generate media coverage.

### Study status

Fieldwork is completed and analysis is in progress.

## Data availability

### Underlying data

At the time of manuscript submission, access to data from the HERO-G study is subject to ethical review by the MRC Unit The Gambia at the London School of Hygiene and Tropical Medicine Ethics Committee ( https://www.mrc.gm/scientific-coordinating-committee/). Study data will be made publicly available no later than July 1, 2021. Requests to access the datasets before that time should be directed to the corresponding author.

### Extended data

Open Science Framework: Gates Open Research Protocol Paper - Extended data. https://doi.org/10.17605/OSF.IO/5PCEX ^10^

This project contains the following extended data:

- LMP.pdf (Last menstrual period form)
- HERO-G Information Sheet and Consent Form.docx (Study information and consent form)
- MatMorbidity.pdf (Maternal morbidity form)

Data are available under the terms of the Creative Commons Attribution 4.0 International license (CC-BY 4.0).
