# Supplementary material for: Identification of nutritionally modifiable hormonal and epigenetic drivers of positive and negative growth deviance in rural African fetuses and infants: Project protocol and cohort description
Source: Gates Open Res. 2020 Feb 24;4:25. [Version 1] doi: 10.12688/gatesopenres.13101.1 (PMC7921526; doi:10.12688/gatesopenres.13101.1)
Supplement: Peer review history

# Peer review: Reviewer response for version 1

**DOI:** 10.21956/gatesopenres.14262.r30297

**Published:** 2021-03-01

**Authors:** Yue-Mei Fan, Laura Adubra

**Affiliations:** 1 Center for Child Health Research, Faculty of Medicine and Health Technology, Tampere University, Tampere, Finland; 2 Center for Child, Adolescent and Maternal Health Research, Faculty of Medicine and Health Technology, Tampere University, Tampere, Finland

This is a well-written and interesting protocol paper to identify nutritionally modifiable hormonal and epigenetic drivers of positive and negative growth deviance in rural African fetuses and infants. The study design is rationale, the methodology is robust. The effort of growth data gathering is thorough for growth curve modelling and fill the gap from previous studies with less frequent sampling and anthropometric measurement.

 As such, our comments are relatively minor. Overall, there are some unclarities in the text that should be addressed.

1. The authors wrote in the Abstract that the data is being used to explore three major mechanistic pathways influencing growth. The third one is – focused measurement of hormones and cytokines that act together in an integrated manner, both *in utero *and after birth, to coordinate patterns of growth with immune activation, inflammation, and nutritional status. However, it’s not mentioned in the paper what hormones and cytokines will be measured.
2. Protocol-> Study measurements-> Pregnancy part:
  -  1 ^st^ paragraph, how MUAC was measured?
  - 2 ^nd^ paragraph, the remaining blood processed and divided into plasma and serum aliquots. Does it mean that some participants had plasma samples, and some had serum samples? If so, why not use the same for every participant? Same for blood processing in child clinic visit.
  - 3 ^rd^ paragraph, suggest changing the order of second and third sentences. Measurement of glucose should be first then record. 
3. Protocol-> Study measurements-> Delivery and week 1 visits part, 4 ^th^ paragraph, 875 mL of supernatant? -> 875 µl
4. Protocol-> Study measurements-> Infancy and early childhood clinic visits part:     - 2 ^nd^ paragraph, “2-mL aliquots of each sample were diluted 20x in 18 mL of …” -> 10x not 20x?
   - 3 ^rd^ paragraph, “additional saline wet preparations were prepared” -> additional saline wet mounts were prepared?
5. Regarding anthropometric measurements during Infancy and early childhood, was there any referral system in place in case of detection of malnutrition signs (for example severely wasted infants detected based on MUAC data). If yes, were these children excluded from the study? 
6. During early infancy and childhood and especially for children who entered the growth faltering protocol, were there any additional data collection on treatment ongoing (for example antibiotic treatment or nutritional supplements received) or data collected on their diet? If yes, is it planned to account for these other factors when conducting analysis to examine the relationship between hormone concentrations and growth? 
7. Table 1 and 2, indicate TEM in footnote.     
8. Figure 2. “Week 20 – Still enrolled 249 and total conducted 124*”, the asterisk was not explained in the chart or text. Also the abbreviation “DNA” should be indicated in footnote.

Is the study design appropriate for the research question?

Yes

Is the rationale for, and objectives of, the study clearly described?

Yes

Are sufficient details of the methods provided to allow replication by others?

Yes

Are the datasets clearly presented in a useable and accessible format?

Yes

Reviewer Expertise:

Child growth, Environmental enteric dysfunction, Malaria, Molecular diagnostic, Public health, Epidemiology, Maternal and child nutrition

We confirm that we have read this submission and believe that we have an appropriate level of expertise to confirm that it is of an acceptable scientific standard.

# Peer review: Reviewer response for version 1

**DOI:** 10.21956/gatesopenres.14262.r30295

**Published:** 2021-01-25

**Authors:** Rukshan Mehta

**Affiliations:** 1 Centre for Global Child Health, The Hospital for Sick Children, Toronto, Canada

This is a well written manuscript which provides robust rationale for the study design to address the relevant research questions. All study protocols are well defined and described with enough information to allow for replication. 

 Only major comment would be that authors provide more details in the last section on 'synthesis' to describe how they intend on using data related to potential predictors i.e. methylation patterns, metabolomic, hormonal and inflammatory markers and intestinal permeability markers to assess growth profiles. The statistical analysis plan is well outlined, but could benefit from some more details related to specific research questions that will address this using the multitude available data. An outline of study hypotheses would also help integrate the data analysis and statistical plan.

Is the study design appropriate for the research question?

Yes

Is the rationale for, and objectives of, the study clearly described?

Yes

Are sufficient details of the methods provided to allow replication by others?

Yes

Are the datasets clearly presented in a useable and accessible format?

Not applicable

Reviewer Expertise:

Maternal and child nutrition, environmental exposures, environmental enteropathy, infant growth, micronutrients, infant gut integrity, targeted metabolomics.

I confirm that I have read this submission and believe that I have an appropriate level of expertise to confirm that it is of an acceptable scientific standard.
